# Evaluating the Effectiveness of Online Assessments and Their Parameters as Predictors of Academic Performance in Undergraduate Medical Education

**DOI:** 10.7759/cureus.62129

**Published:** 2024-06-11

**Authors:** Palani Selvam Mohanraj, Arani Das, Vinoth Rajendran, Niranjan Gopal, Kothandan Saravanan, Yuvaraj Balan

**Affiliations:** 1 Biochemistry, All India Institute of Medical Sciences, Gorakhpur, Gorakhpur, IND; 2 Physiology, All India Institute of Medical Sciences, Gorakhpur, Gorakhpur, IND; 3 Community Medicine & Family Medicine, All India Institute of Medical Sciences, Gorakhpur, Gorakhpur, IND; 4 Biochemistry, All India Institute of Medical Sciences, Nagpur, Nagpur, IND; 5 Biochemistry, Postgraduate Institute of Medical Education and Research Satellite Centre Sangrur, Sangrur, IND; 6 Biochemistry, All India Institute of Medical Sciences, Madurai, Madurai, IND

**Keywords:** student perspectives, predictive validity, multiple-choice questions, academic performance, medical education, online assessments

## Abstract

Background and objectives

Considering the increasing utilization of online educational tools in medical education, it is essential to evaluate the reliability and validity of online assessments to accurately assess student proficiency and predict academic success. This study investigated the predictive efficacy of different online assessment methods in comparison to standard offline methods within the medical educational setting.

Methods

This study utilized a mixed-methods crossover design, involving 125 first-year medical students who were randomly assigned to either online or traditional examinations. The students then crossed over to the other type of assessment. The assessments consisted of multiple-choice questions (MCQs), viva voce, objective structured clinical examinations, and written theory examinations. Quantitative data on results, finishing times, and academic metrics were analyzed, along with qualitative data from student interviews exploring perceptions of each format.

Results

The online MCQs had the highest average scores and a moderately positive correlation with performance on the theory examination (r=0.326). Regression models indicated that online and offline MCQs were moderate positive predictors of theoretical marks (R^2^=0.106 and 0.107, respectively). Qualitative responses emphasized advantages such as flexibility and accessibility for online examinations but also concerns regarding technological challenges, interaction, and integrity compared to traditional formats.

Conclusions

Online MCQ assessments showed promise as indicators of medical student academic performance. However, additional online forms require improvement to match conventional assessments reliably. As medical education involves digital technologies, cautious implementation of online evaluations substantiated by further research is needed to preserve educational quality standards.

## Introduction

As educational systems progressively integrate digital technologies, the highly specific domain of medical education faces unique challenges and opportunities. The transition from traditional pedagogical frameworks to digital platforms needs a comprehensive reevaluation of assessment procedures to ensure they fulfill the stringent standards required for successfully training future healthcare practitioners. This study emphasizes investigating the usefulness of online assessment tools in predicting medical undergraduates' academic performance, as well as scrutinizing the dependability and capability of various online formats to estimate student competency professionally.

Historically, medical training has mainly relied on personal interactions and practical learning to measure student knowledge and clinical skills efficiently. However, the rise of learning management systems and an extensive range of digital assessment technologies, ranging from multiple-choice questions (MCQs) to sophisticated virtual simulations, poses challenges regarding their capacity to match the outcomes of traditional examinations. Preliminary research reveals a wide range of success in these digital tools compared to conventional approaches, particularly in the field of basic science within medical education [1-4].

Contemporary research efforts, such as those by Kara et al. [5] and Saiyad et al. [6], reflect an evolving understanding of online educational practices, underscoring that while digital platforms facilitate accessibility and potential cost reductions, they demand meticulous implementation to maintain educational quality. Most importantly, the integrity of online assessments is critical; these tools must precisely evaluate knowledge and promote its application within clinical settings, a vital part of medical training where patient safety and good clinical practice are at stake.

Recent data reveals a significant growth in the implementation of online assessments in medical teaching institutions globally. A global study by Dedeilia et al. showed that 65% of medical schools in Europe and Asia utilized digital platforms for both formative and summative evaluations, driven by the COVID-19 pandemic [7]. These platforms comprise a variety of technologies, ranging from MCQs and virtual patient simulations to interactive case studies and oral examinations conducted via video conferencing [8]. In addition, a study by Sandhu and de Wolf [9] indicated that 68% of medical schools globally had integrated digital assessment tools as a response to the pandemic, suggesting a considerable shift toward online education in medical training. The rising use of these technologies reflects a broader trend toward digital transformation in medical education, seeking to promote accessibility, flexibility, and efficiency in measuring student competency.

Both online and offline assessments come with their own set of benefits and drawbacks. Online assessments offer the benefits of convenience, flexibility, and scalability, making it easier for students to take tests from any place and for teachers to manage large batches. They also provide instant feedback and the ability to include multimedia elements, which might improve the assessment process. However, they pose challenges such as technical problems, potential cheating, and a lack of personal interaction, which may negatively impact the reliability and integrity of the assessments. In contrast, traditional offline examinations are well-established and can be more effective in assessing practical abilities through hands-on experiences. They offer a controlled environment that discourages cheating and allows for direct observation by teachers. However, they are less flexible, are often more time-consuming, and can be resource-intensive, requiring physical space and materials.

This study intends to critically analyze the reliability and validity of online assessments in comparison to traditional approaches for predicting academic performance among medical students. It attempts to determine which online assessment formats - MCQs, oral examinations, and practical simulations - are most effective and demonstrate why this is the case. Additionally, the study will analyze perspectives of students and staff toward online evaluations to determine perceived strengths and limitations within a medical educational framework.

The necessity of this study is highlighted by the rapid universal shift toward online education, prompted by the need for educational institutions to ensure that this change does not undermine the quality of medical training. As the medical sector progressively utilizes digital technologies and telemedicine, it is vital that new medical graduates are efficient in employing technology in their profession. Furthermore, the insights gathered might contribute to the development of innovative assessment methods that improve educational outcomes and generate more competent medical professionals, aligning with the demands of a dynamically expanding healthcare environment.

## Materials and methods

Study design

This research utilized a mixed-methods crossover study design to fully investigate the effectiveness of various online assessment tools in predicting the academic performance of medical undergraduates. The mixed-methods approach combined both quantitative and qualitative components to provide a comprehensive evaluation. The quantitative component aimed to compare the efficacy of online versus offline assessments through a crossover design where each participant acted as their own control. The qualitative component sought to explore the perceptions and experiences of students regarding online and traditional assessments. The crossover design included randomization of participants to minimize selection bias and a crossover period where participants switched from their initial assessment method to the other after a predetermined interval. This study was conducted in the Department of Biochemistry, All India Institute of Medical Sciences, Gorakhpur, Gorakhpur, Uttar Pradesh, India, in collaboration with the Department of Physiology, and both quantitative and qualitative research methodologies were applied to give a comprehensive look at the efficacy of online versus traditional assessments.

Study participants

The study included 125 medical students pursuing first-year MBBS (phase 1) at the All India Institute of Medical Sciences, Gorakhpur. These students were randomly allocated to one of two groups to ensure an equal distribution based on prior academic performance, age, and gender, thereby minimizing any biases associated with learner characteristics. No formal sample size calculation was conducted for this study. However, a post-analysis power calculation was performed to ensure adequate statistical power.

Study interventions

Initially, group A completed online tests, whereas group B engaged in traditional offline assessments. The examinations included MCQs, viva voce (oral examinations), and objective structured practical examinations (OSPEs). The assessment details, including topics/units from the curriculum, scoring rubrics, weightages, and total marks distribution, are provided in the Appendices as a supplementary file. After a predefined period, the groups switched modalities, with group A doing traditional offline assessments and group B taking online assessments. This crossover approach allowed each student to experience both types of assessment under similar conditions. The students were provided a demonstration and discussion regarding the whole assessment process (online and offline) during the orientation program. Online mock tests were conducted with walkthroughs for orientation to the interface and to clarify any doubts about using the online platform well ahead of the actual assessment.

Data collection

Demographic data of the study participants such as age, gender, college entrance scores were collected. Quantitative data were obtained using the scores obtained from both the online and traditional offline assessments. Additional data regarding the time taken to complete each assessment and the students' performance statistics offered by the online platform were also obtained. Qualitative data were obtained via structured interviews and focus groups with participants after they completed both stages of the study, seeking to capture their perceptions of the fairness, clarity, and engagement level of each assessment type. Online assessments were administered using a secure, web-based educational infrastructure such as Google Workspace for Education, Google Forms, Google Meet, and Testmoz, which enabled real-time data collecting and analysis. These technologies also permitted the administration of viva voce and OSPEs in a virtual setting, imitating the conditions of offline assessments as nearly as possible.

Statistical analysis

Quantitative data were examined using R statistical software (R Foundation for Statistical Computing, Vienna, Austria) to conduct paired t-tests to compare the means of scores between the two types of assessments across both groups. The normality of continuous data was assessed using the Kolmogorov-Smirnov test. Correlation and regression studies were undertaken to explore the correlations between assessment scores and academic performance in subsequent traditional examinations. The analysis was carried out at 5% level of significance, and p<0.05 was considered statistically significant.

Qualitative analysis

Thematic analysis was performed to evaluate the qualitative data obtained from interviews and focus groups. This investigation discovered common themes and trends involving students' experiences and views of online versus traditional assessments.

Ethical considerations

Ethical approval was obtained from the Institute Ethics Committee (Human Studies), All India Institute of Medical Sciences, Gorakhpur, under reference number IHEC/AIIMS-GKP/BMR/98/2022 prior to the initiation of the study. Informed consent was obtained from all participants, ensuring they were fully aware of the study’s nature, the confidentiality of their data, and their right to withdraw at any moment without any repercussions.

## Results

The descriptive statistics summarized in Table 1 showed variations in the mean scores across different assessment types.

**Table 1:** Descriptive statistics of the study variables MCQ, multiple-choice question; OSPE, objective structured practical examination

	Mean ± SD	Median	Min	Max
Online MCQ score	65.71 ± 12.96	68	24	90
Offline MCQ score	56.24 ± 15.12	52	20	88
Online viva voce score	54.68 ± 9.93	55	25	75
Online OSPE score	56.44 ± 7.64	56.88	39.38	74.38
Gold standard test score	49.79 ± 9.81	51.53	13.75	68.15

**Figure 1:**
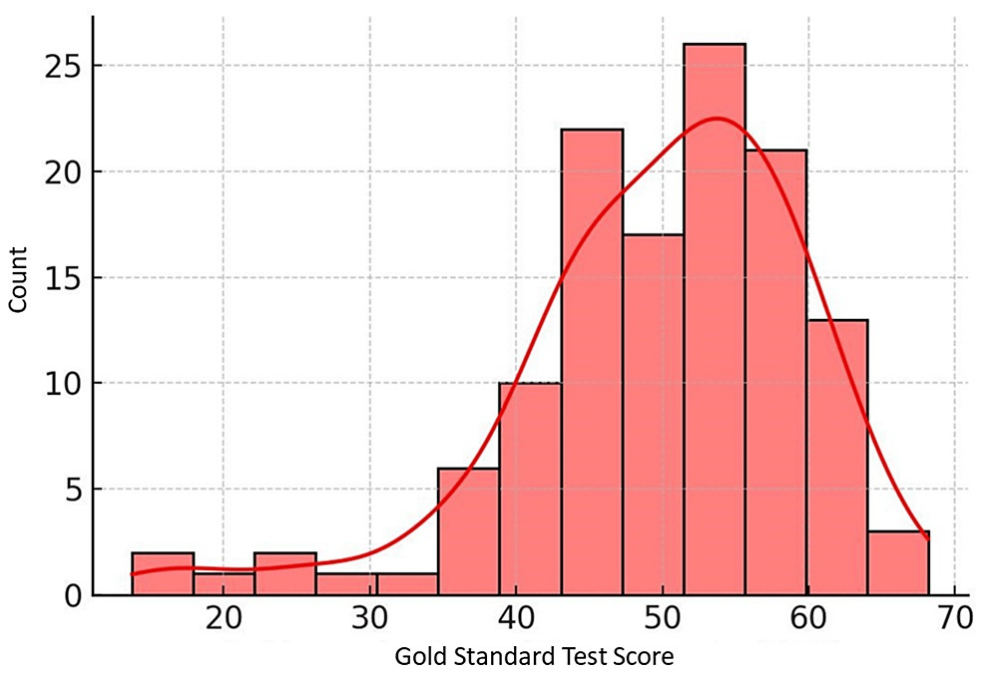
Distribution of gold standard test scores (theory marks)

**Figure 2:**
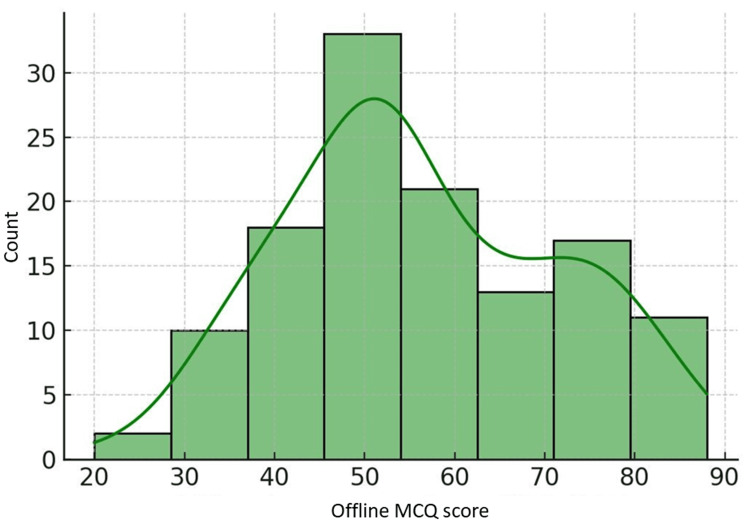
Distribution of offline MCQ scores MCQ, multiple-choice question

**Figure 3:**
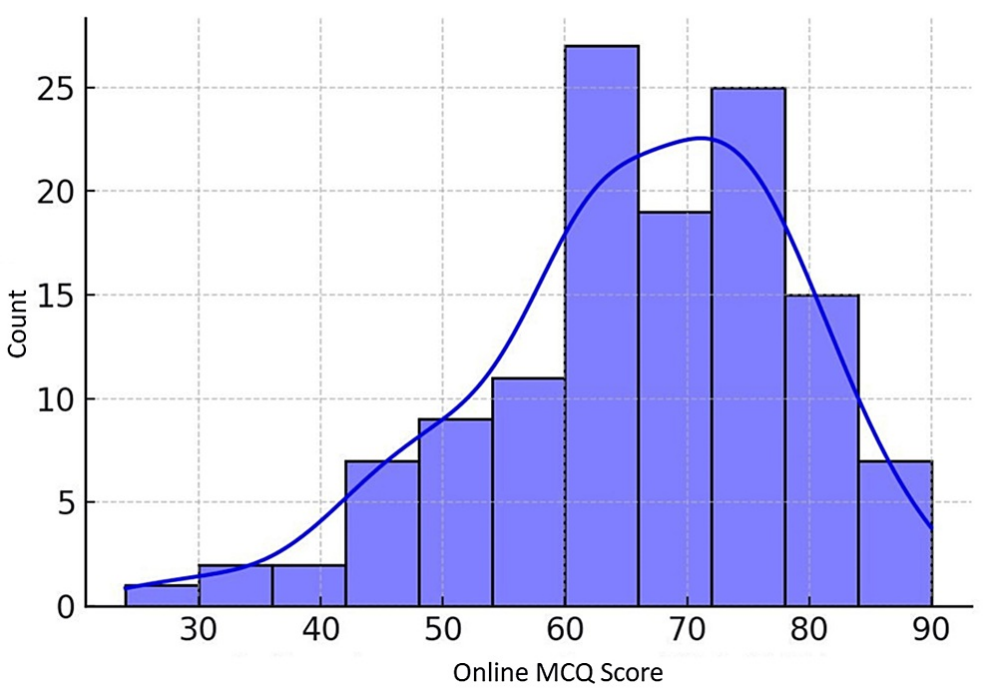
Distribution of online MCQ scores MCQ, multiple-choice question

Online MCQ assessments had the highest mean score (65.71) among the online assessments, followed by OSPE (56.44) and viva voce (54.68). The offline MCQ assessment mean (56.24) was comparable to the online OSPE and viva voce means. However, the gold standard written examination mean (49.79) was noticeably lower than the means for most online and offline assessments. The distributions visualized in Figures 1-3 highlighted a slight left skew for online MCQs and more spread for offline MCQs.

Inferential statistics using paired t-tests (Table 2) revealed statistically significant differences between online and offline MCQ scores (t=5.57, p<0.001), online viva voce and theory marks (t=5.38, p<0.001), and online OSPE/OSCE and theory marks (t=8.66, p<0.001).

**Table 2:** Statistical comparison between types of assessment MCQ, multiple-choice question; OSPE, objective structured practical examination

Assessment type	T-Statistic	p-Value
Online MCQ vs. offline MCQ score	5.57	<0.0001
Online viva voce vs. gold standard test score	5.38	<0.0001
Online OSPE vs. gold standard test score	8.66	<0.0001

Correlation analysis (Table 3) showed moderate positive correlations between online MCQs (r=0.326, p=0.00021) as well as between offline MCQs (r=0.327, p=0.0002) and theory marks, suggesting their potential as predictors of academic performance.

**Table 3:** Correlation matrix of study variables MCQ, multiple-choice question; OSPE, objective structured practical examination; NEET, National Eligibility-cum-Entrance Test

	Online MCQ score	offline MCQ score	Online viva voce score	Online OSPE score	Gold standard test score	NEET score
Online MCQ score	1.00	0.09	0.43	0.41	0.33	-0.17
Offline MCQ score	0.09	1.00	0.50	0.40	0.33	0.00
Online viva voce score	0.43	0.50	1.00	0.70	0.47	-0.01
Online OSPE score	0.41	0.40	0.70	1.00	0.54	-0.03
Gold standard test score	0.33	0.33	0.47	0.54	1.00	-0.09
NEET score	-0.17	0.00	-0.01	-0.03	-0.09	1.00

In contrast, college admission scores exhibited a very weak, non-significant negative correlation (r=-0.09, p=0.316) with theory marks.

Single predictor regression models revealed online MCQs (R^2^=0.106) and offline MCQs (R^2^=0.107) as moderate positive predictors of theory marks. The combined multiple regression model using all three predictors showed a slight improvement (adjusted R^2^=0.092), with online and offline MCQs positively predicting theory marks, while college admission scores had a slight negative effect.

Sentiment analysis indicated generally positive views toward online assessment advantages but more critical perspectives on disadvantages. Key themes from the qualitative data summarized in Table 4 highlighted time efficiency, accessibility, and flexibility as advantages, while technical issues, lack of interaction, and cheating concerns were noted as disadvantages. Suggestions focused on security enhancements, interactive elements, and technical support.

**Table 4:** Thematic analysis

Theme	Number of mentions	Examples
Security enhancements	20	Use of proctoring software
Interactive elements	15	Real-time Q&A sessions
Technical support	25	24/7 tech support

**Figure 4:**
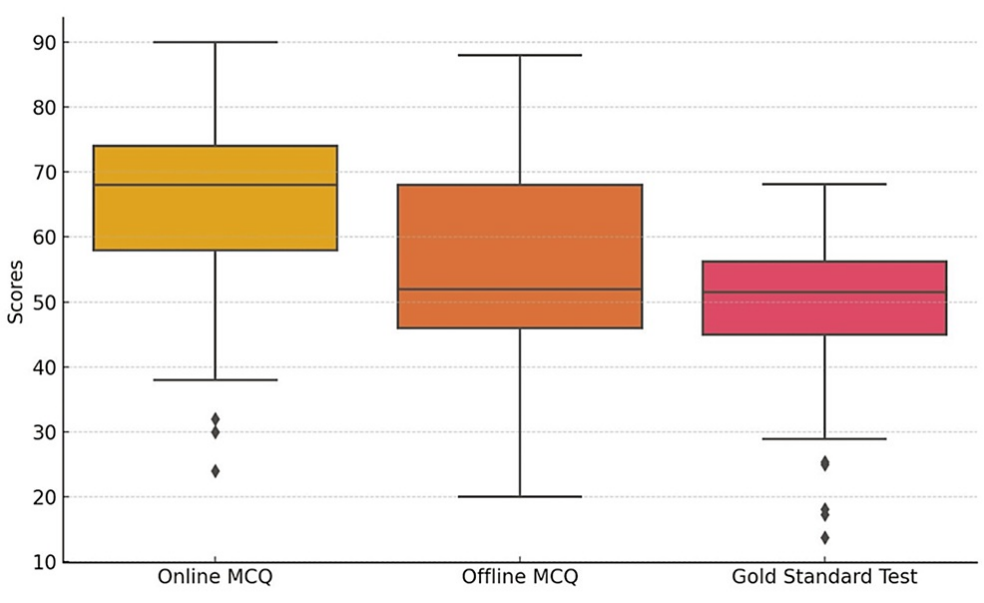
Box plot of assessment scores MCQ, multiple-choice question

Finally, the box plots in Figure 4 revealed outliers in theory marks and offline MCQs.

**Figure 5:**
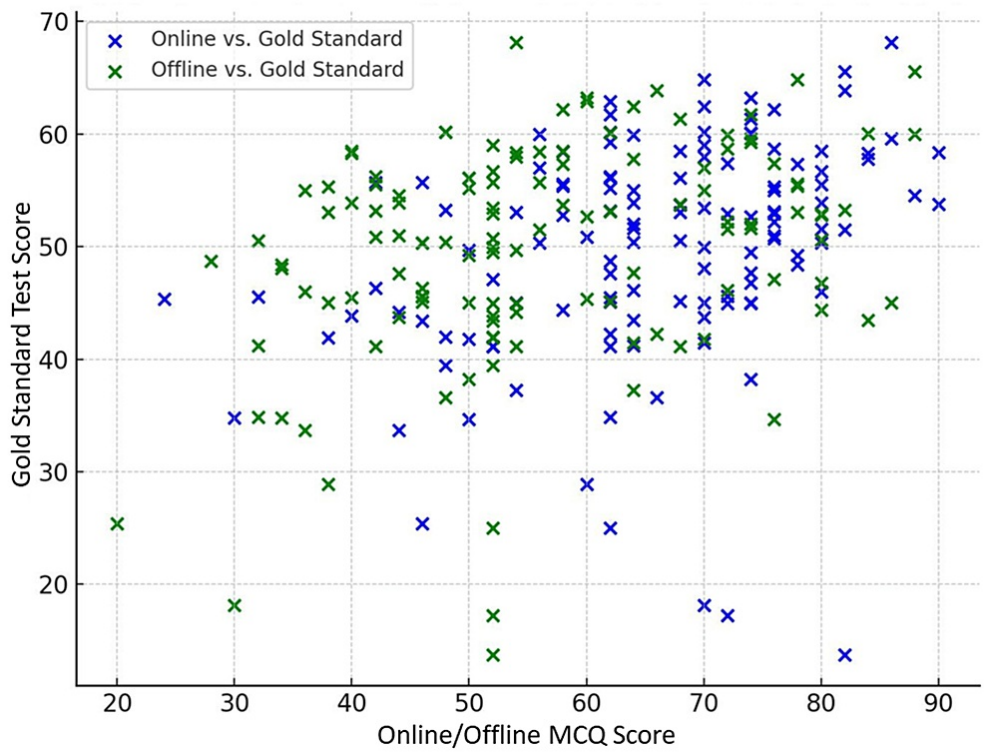
Scatter plot of online/offline MCQ score vs. gold standard test score MCQ, multiple-choice question

Scatter plots in Figure 5 visually reinforced the moderate positive relationships between online/offline MCQs and theory marks, aligning with the correlation/regression findings.

Overall, these results suggest online assessments, particularly MCQs, have potential to predict academic performance comparably to traditional assessments. However, variations across assessment modes underscore the need for carefully integrating and improving online assessments for reliable evaluation.

## Discussion

This study assessed the efficacy of online assessments compared to conventional offline assessments in predicting the academic performance of medical students. The findings suggested that online MCQ assessments are superior to other online formats, conventional offline MCQs, and theoretical examinations in terms of average scores. The statistical analysis showed strong positive association between the scores of both online and offline MCQs and the performance in offline theory examinations. This suggests that these scores have the potential to be useful in estimating academic performance.

While the correlation analysis did reveal moderate positive correlations of online and offline MCQ with theory examination scores (r=0.326 and 0.327, respectively), indicating MCQs are positive predictors of theory performance, the descriptive statistics showed a lower mean for the theory examination (49.79) compared to MCQ means (65.71 online, 56.24 offline). This apparent discrepancy can be explained by the fact that correlation measures the strength of a linear relationship between variables, not the absolute values themselves. A positive correlation simply means that as one variable increases, the other tends to increase as well, regardless of their actual score values. Therefore, in summary, while MCQ scores did exhibit a positive predictive relationship with theory performance based on correlation, the absolute MCQ mean scores were still statistically significantly higher than theory examination means, likely due to inherent differences in assessment difficulty, design, and skills tested. The correlation analysis (Table 3) also revealed a very weak negative correlation (r=-0.09, p=0.316) between students' National Eligibility-cum-Entrance Test (NEET) scores and their marks in the theory examination conducted during the first year of the medical program. A negative correlation coefficient indicates that as one variable increases (NEET scores), the other variable (theory marks) tends to decrease, and vice versa. However, it is important to note that the correlation value of -0.09 is extremely low, suggesting a very weak negative relationship. This negative correlation, though weak, could potentially signify that entrance examination scores alone may not be an accurate predictor of a student's theoretical knowledge and performance once they progress into the professional medical curriculum. The qualitative data revealed advantages of online examinations, such as improved efficiency and accessibility. However, it also identified concerns regarding technological difficulties, limited engagement, and potential security risks when compared to traditional offline examinations. In summary, the study confirms the ability of online MCQ examinations to predict the performance of medical students. However, it also highlights the necessity of improving the planning and execution of online assessments.

The results of our study are consistent with the prior research conducted by Fatima et al., which provides evidence for the efficacy of online assessments in assessing fundamental scientific knowledge [1]. Additionally, insights from studies by Kara et al., Saiyad et al., and Shen et al. underscore the importance of tailored support, good online teaching practices, and structured cooperative activities in online settings [5,6,10]. The study is consistent with the perspectives of Poleneni and Chandrupatla regarding the complementary role of technology in improving conventional teaching methods and offering valuable formative feedback through online assessments [11]. Furthermore, the study verifies the findings of Wilkinson et al. and Zakaria et al., indicating the substantial associations and predictive capability of online MCQs for theoretical examination scores compared to alternative online formats [12,13].

The study's focus on well-designed MCQs is consistent with earlier research that has shown their effectiveness in assessing higher-order cognitive abilities when they are properly constructed [14,15]. The qualitative feedback also reinforces the advantages mentioned in previous research, such as improved adaptability, accessibility, and the possibility of reducing costs [16,17]. However, it also raises concerns regarding technological challenges and potential risks to the validity of results, such as cheating [16,18]. It is important to highlight that while this study focuses on predictive validity, establishing other degrees of validity and reliability in online evaluations is crucial [19]. Future studies should focus on exploring online assessment design ideas based on validity frameworks and learning concepts.

One limitation of the study was the small number of participants from a single institution; however, the crossover design boosted the quality of the research. To improve generalizability, larger multi-center trials with different samples are recommended. Future research could also investigate the impact of multimedia, simulations, and adaptive question paths on validity and cognitive engagement in online examinations compared to traditional techniques. Exploring innovative hybrid methods that combine the capabilities of online and offline evaluations may further boost learning and assessment processes in medical education.

## Conclusions

This study emphasizes the potential of online MCQs as predictors of medical student performance, while indicating areas for improvement in alternative assessment formats. Adapting online assessments based on current research findings will be essential for maintaining educational standards and maximizing their benefits as medical education continues to evolve.

## Appendices

Supplementary File: Assessment Details

1. Topics/Units from Curriculum Assessed

Multiple-Choice Questions (MCQs):

·         Biochemistry: Biomolecules, Enzymes, Bioenergetics, Nutritional Biochemistry

·         Physiology: Blood, Nerve-Muscle Physiology, Respiratory System, Cardiovascular System

Viva Voce:

·         Biochemistry: Biomolecules, Enzymes, Nutritional Biochemistry

·         Physiology: Blood, Nerve-Muscle Physiology

OSPE:

·         Biochemistry: Practical experiments on Biomolecules, Enzyme assays

·         Physiology: Hematology experiments, Respiratory/Cardiovascular system demonstrations

Theory Examination:

·         Biochemistry: Entire portion covered in first-year

·         Physiology: Entire portion covered in first-year

2. Scoring Rubrics

MCQs: 1 mark for correct, 0 for incorrect (no negative marking)

Viva: Scoring rubric - 0-5 scale based on depth of knowledge, explanation, and reasoning ability

OSPE: Checklist-based scoring rubrics specific to each station, ranging from 0-10 marks

Theory: Subjective marking schemes

·         Very Short Answer Questions (2-3 marks based on key points)

·         Short Answer Questions (3-5 marks based on key points)

·         Long Answer Questions (8-10 marks based on comprehensiveness)

3. Weightages

Online Assessments:

·         MCQs: 60%

·         Viva: 20%

·         OSPE: 20%

Offline Assessments:

·         MCQs: 60%

·         Viva: 20%

·         OSPE: 20%

4. Total Marks Distribution

Online Assessments:

·         MCQs: 60 marks

·         Viva: 20 marks

·         OSPE: 20 marks

·         Total: 100 marks

Offline Assessments:

·         MCQs: 60 marks

·         Viva: 20 marks

·         OSPE: 20 marks

·         Total: 100 marks
